# Directly grown two dimensional In_2_S_3_ nanoflakes via one-step solvothermal method: Material properties on In_2_S_3_ and performance data for supercapacitors

**DOI:** 10.1016/j.dib.2020.106272

**Published:** 2020-09-03

**Authors:** Niraj Kumar, Dhananjay Mishra, Seung Yeob Kim, Taehui Na, Sung Hun Jin

**Affiliations:** Department of Electronic Engineering, Incheon National University, Incheon 21999, Republic of Korea

**Keywords:** Solvothermal process, Layered structure, Supercapacitor, Indium sulfide and energy storage

## Abstract

Herein, the material structural properties such as phase, morphology, chemical composition, and surface area for In_2_S_3_ nanoflakes, synthesized by a one-step solvothermal method, are studied [1]. The comparative electrochemical performance data of indium based electrode material is presented to establish the practical suitability of prepared In_2_S_3_ electrode material. Device demonstration of fabricated solid-state supercapacitor device on different time frames set performance level demonstration of current work and suggest a potential candidate for next-generation energy storage electrode material.

## Specifications Table

**Table:** 

Subject	Physics, Electronic Engineering
Specific subject area	Materials Science, Supercapacitor
Type of data	Table Figure
How data were acquired	X-ray diffraction (XRD), Rigaku, smart Lab, the 2-theta scan range of 20ᵒ−80ᵒ using Cu _Kα_ radiation (λ = 1.54 Å) irradiation) Grazing incidence X-ray Diffraction (GIXRD), fixed incidence- angle (α) of 0.5˚, the 2-theta scan range of 20ᵒ−80ᵒ Field emission scanning electron microscope (FESEM, JOEL, JSM_7800 F, operating voltage =15 kV and working distance (10 mm)) Energy dispersive spectroscopy (EDS, JEOL, EDS-7800F) measurements of the synthesized nanoparticle were performed to quantify the chemical composition (operating voltage =15 kV and working distance (10 mm)) FTIR, FT-IR Microscope: HYPERION 2000, range: 4000 - 800 −1 TEM, high-resolution TEM (HRTEM), atomic-resolution high-angle annular dark-field (HAADF) were recorded using an FEI/Talos F200X Specific surface area (Brunauere Emmette Teller (BET)) and pore size distribution (Barrete Joyere Halenda (BJH)), by using N2 adsorption-desorption isotherms by Tristar, ASAP 2020 / MICROMERITICS. All electrochemical measuremants, by Compactstat.h IVIUM Technologies electrochemical workstation
Data format	Raw and analysed
Parameters for data collection	X-ray diffraction (XRD, Rigaku, smart Lab, the 2-theta scan range of 20ᵒ−80ᵒ using Cu _Kα_ radiation (λ = 1.54 Å) irradiation) Field emission scanning electron microscope (FESEM, JOEL, JSM_7800 F, operating voltage =15 kV and working distance 10 mm) Energy dispersive spectroscopy (EDS, JEOL, EDS-7800F) measurements of the synthesized nanoparticle were performed to quantify the chemical composition (operating voltage =15 kV and working distance (10 mm)) FTIR, FT-IR Microscope: HYPERION 2000, range: 4000 - 800 −1 TEM, high-resolution TEM (HRTEM), atomic-resolution high-angle annular dark-field (HAADF) were recorded to understand atomic layer level structure Specific surface area (Brunauere Emmette Teller (BET)) and pore size distribution (Barrete Joyere Halenda (BJH)), by using N2 adsorption-desorption isotherms by Tristar, ASAP 2020 / MICROMERITICS. Electrochemical measuremants – cyclic voltammetry (CV) and Electrochemical Impedance Spectroscopy (EIS) by Compactstat.h IVIUM Technologies electrochemical workstation
Description of data collection	Growth of Sponge-Like In_2_S_3_ nanoflakes via a solvothermal process
Data source location	Incheon National University, Incheon 22,012, Korea
Data accessibility	The data are with this article
Related research article	Niraj Kumar, Dhananjay Mishra, Seung Yeob Kim, Taehui Na*, and Sung Hun Jin* “Two Dimensional, Sponge-Like In_2_S_3_ Nanoflakes Aligned on Nickel Foam via One-Pot Solvothermal Growth and Their Application toward High Performance Supercapacitors” Materials Letters For a co-submission research article (doi.org/10.1016/j.matlet.2020.128467)

## Value of the Data

• The study on structure and morphology of directly grown In2S3 nanoflakes via one-step solvothermal method can be useful to develop design rules for implementing effective electrode materials for highperformance supercapacitor devices.
• Both obtained low-resolution SEM, TEM images, and adsorption/desorption BET data demonstrate layered structure, uniform distribution over nickel foam substrate, and porous material structure for favourable electrochemical properties for supercapacitors.
• Comparison of previously reported indium-based electrode materials in terms of electrochemical characteristics with our current work, identifying that the suitability of current work for supercapacitor application is promising.
• LED demonstration with our fabricated SSC device on different time frames (0 to 150 s) shows real-time high performance of the constructed device.

## 1 Data Description

**Fig. 1:**
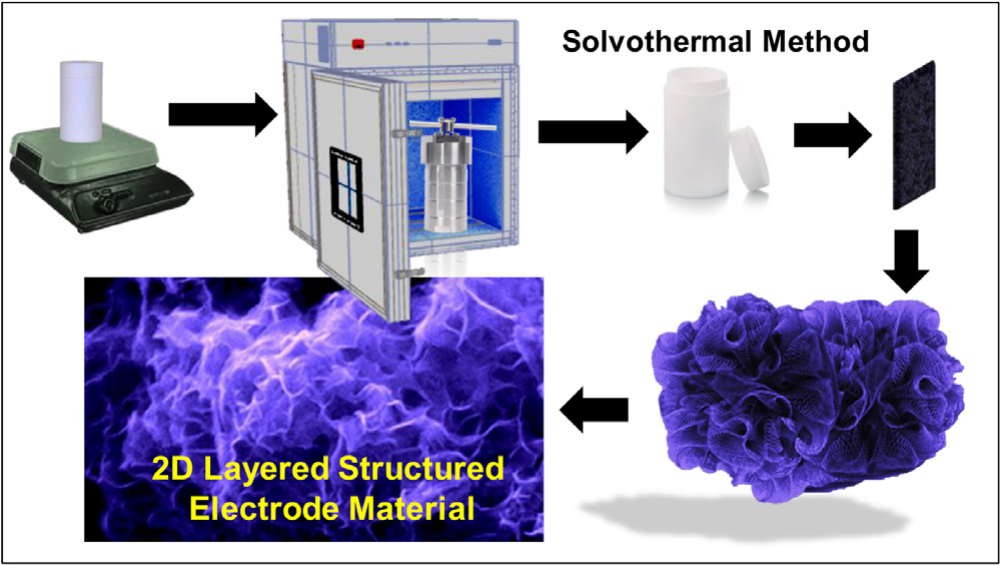
Experimental scheme and possible layered microstructure formation by solvothermal method.

Two-dimensional (2D) direct grown layered In_2_S_3_ nanoflakes electrode material is synthesized by facile one-step solvothermal method [1]. Fig. 1 is showing the facile process flow for the preparation of layered nanoflake In_2_S_3_ electrode material via a one-step solvothermal method.

## 2 Reaction Process of 2D Layered In_2_S_3_ Material

Thiourea is used as sulfur-rich precursor to synthesize indium sulfide material. The high molar ratio of thiourea (1:5 molar ratio) was taken to provide sulfur-rich environment for metal sulfide synthesis [2]. The reaction mechanism for In_2_S_3_ synthesis can be speculated as to the following reaction:

(1) $$3In{\left(NO_{3}\right)}_{3}\;+\;2CH_{4}N_{2}S\;+4{\left(OH\right)}^{-}\to \;4NH_{3}+2CO_{2}+9{\left(NO_{3}\right)}^{-}+In_{3\;}S_{2}$$

To evaluate the effect of slovothermal reaction on Ni foam substrate, we perform grazing incidence X-ray Diffraction (GIXRD) characterization with a fixed incidence- angle (α) of 0.5˚ for three samples, direct grown In_2_S_3_ on nickel foam, bare nickel foam substrate (NF1) and nickel foam after solvothermal reaction without adding indium precursor (NF2).

**Fig. 2:**
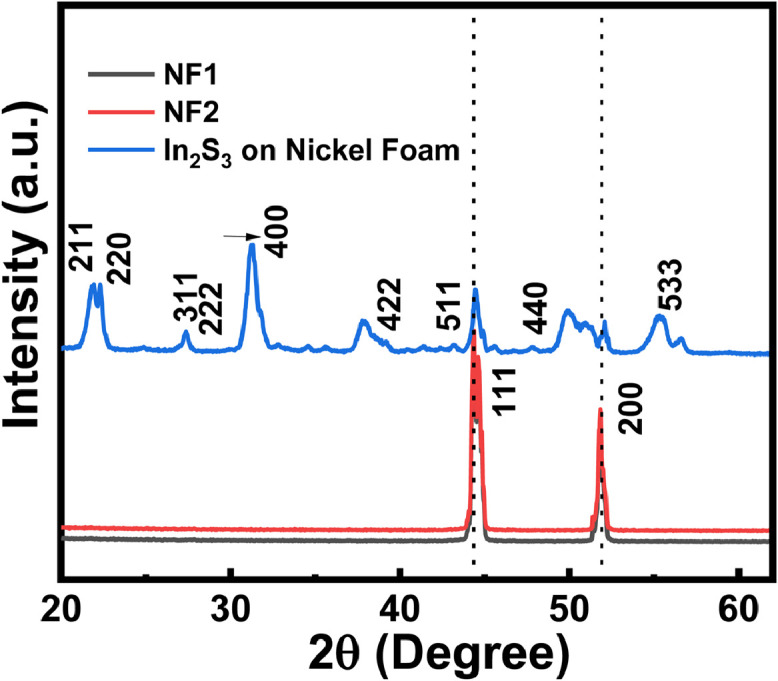
XRD patterns of bare Ni foams, Ni foam processed without indium precursor, and directly grown In_2_S_3_ on Ni substrate. NF1 (or NF2) stands for Ni foam without (or with) indium precursor, respectively.

We kept nickel foam substrate for solvothermal reaction with the same experimental condition, without adding indium precursor (i.e., indium nitrate) and obtained nickel foam nominated as NF2. XRD spectra for NF2 in Fig. S2 shows a similar peak with slightly diminished peak intensity as compared with that of NF1, identifying that Ni foam is not sulfided after the solvothermal reaction. XRD spectrum for the prepared thin film sample In2S3 on nickel foam is indexed with miller indices, with standard ICDD pattern 32–0456, corresponding to the In_2_S_3_ cubic structure (Fig. 2) .

**Fig. 3:**
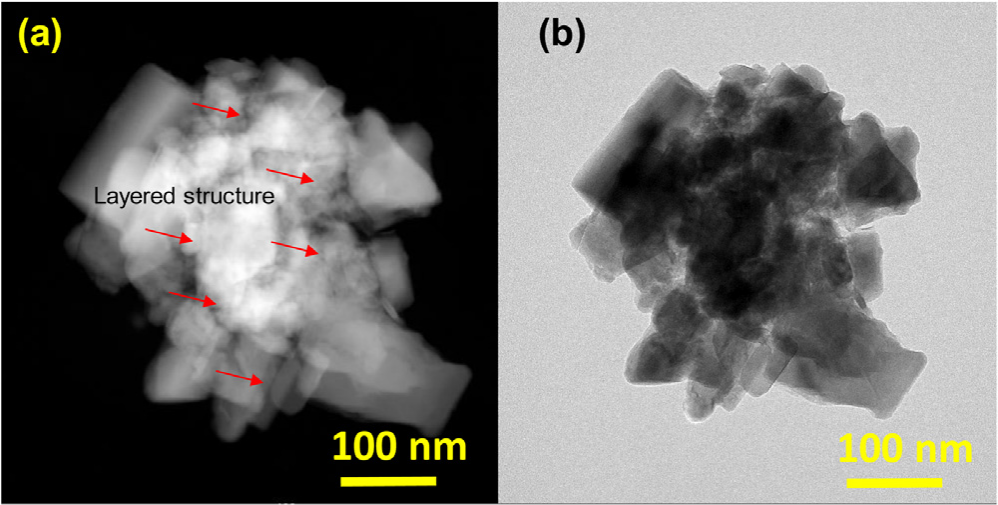
(a) dark filed of TEM and (b) Bright TEM field images.

HRTEM analysis was accomplished to evaluate the atomic level formation and structural study [1]. Fig. 3a and b are showing bright and dark field TEM images. Layered distribution can be observed clearly on completely material distribution. Red color arrow in Fig. 3a is showing layered structure on distributed material.

**Fig. 4:**
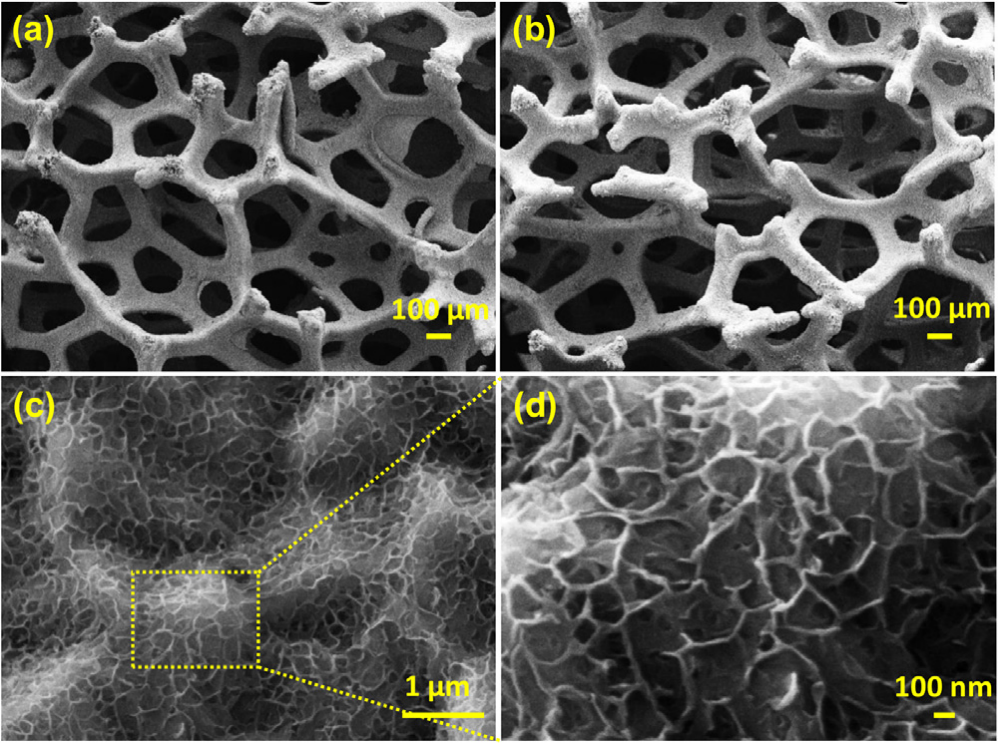
Wide range of FESEM image of layered In_2_S_3_ on different magnifications.

Wide range FESEM images of prepared In_2_S_3_ material on nickel foam at different magnification (high and low) show the clear distribution of formed uniform layered structure. Uniformity and nanoflake orientation is confirmed from displayed wide range FESEM images (Fig. 4a-d)

**Fig. 5:**
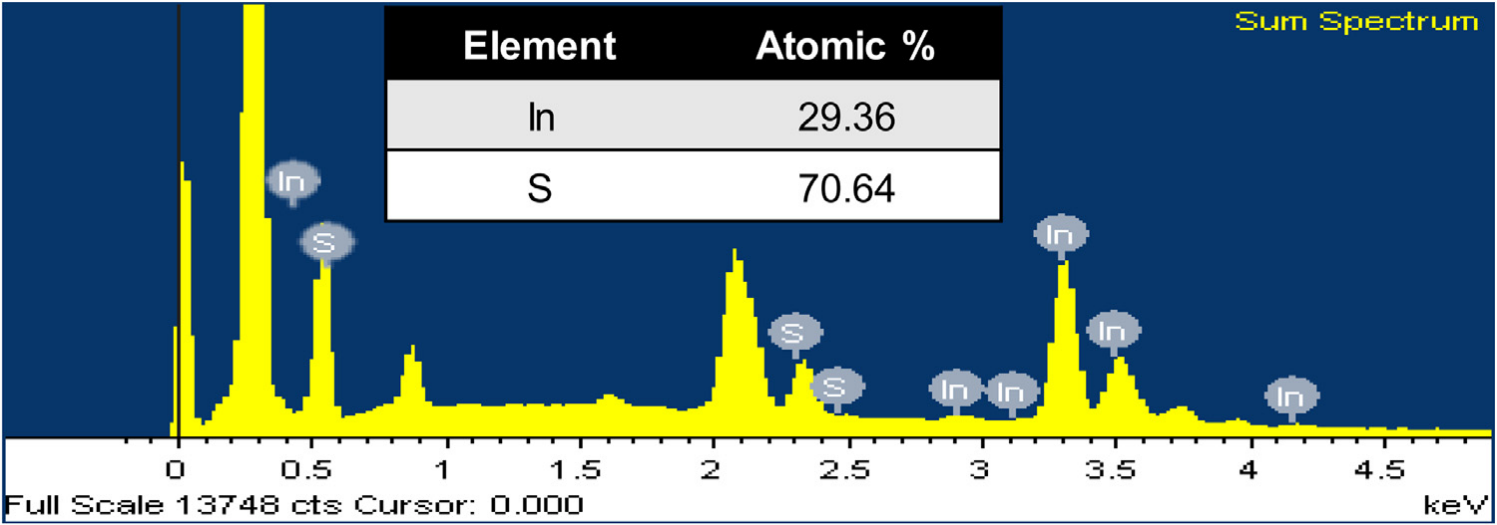
EDX spectrum (inset atomic value%) of sample In_2_S_3_.

In Fig. 5, the EDAX spectrum demonstrates the chemical element distribution and elemental composition. This spectrum shows the presence of constitutes element indium and sulfur in the material. Extra peaks indicate coated platinum and some available oxygen elements. Presence of atomic% of Indium and sulfur element confirmed the prepared In_2_S_3_ elemental composition.

**Fig. 6:**
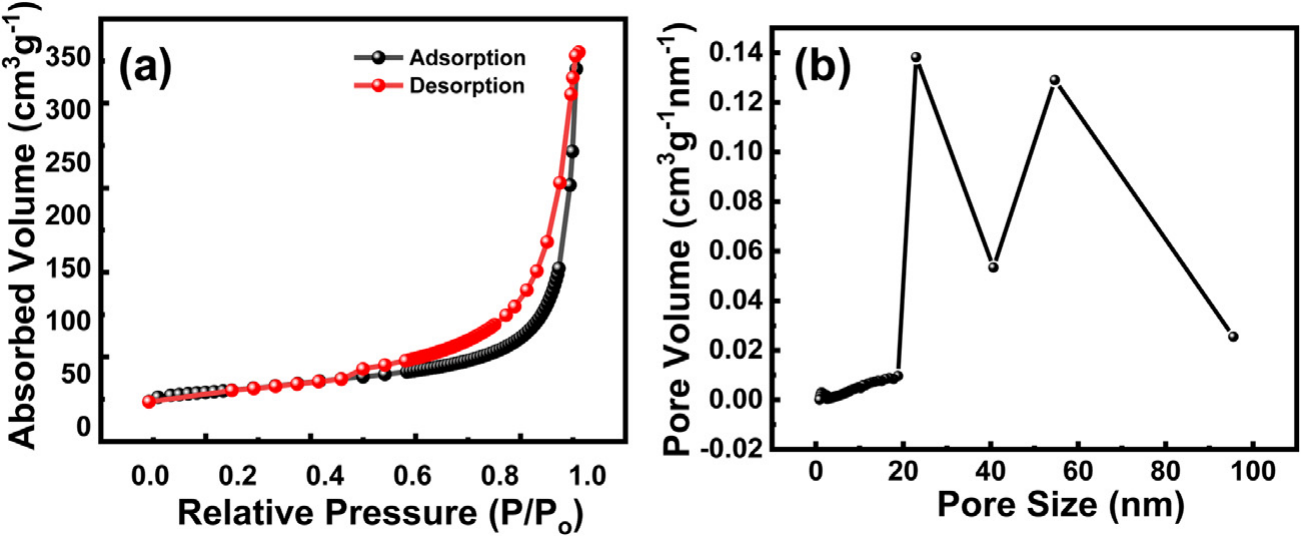
(a) Nitrogen adsorption/desorption isotherm and (b) Pore-size distribution spectrum of electrode material.

Specific surface area and micropores/mesopores in the prepared sample were evaluated by the N2 adsorption/desorption isotherm test and obtain spectrum is shown in Fig. 6a. Observed hysteresis loop in the range of relative pressure (P/Po) from 0.45 to 0.98, indicates type IV isotherm and presence of mesopores. The obtained specific surface area of prepared layered material is 88.50 m^2^g^−1^. Mesopores/macropores pore size distribution is shown in Fig. 6b, which is derived from the desorption branch isotherm data by BJH method. The pore-size distribution spectrum indicates mesopores volume is more distributed in the prepared sample.

## 3 Electrochemical Measurements Details-

From CV curves, specific capacitance (C_s_) is measured by the following equation:

(2) $$C_{s}=\frac{1}{mv\left(V_{2}-V_{1}\right)}\int_{V_{a}}^{V_{C}}I\left(V\right)\text{dV}$$

Here ‘m’ correspond to deposited electrode material mass on NF substrate and measured by mass difference method, ‘v’ is scan rate, I (V) is response current and (V_2_ – V_1_) is potential window taken for CV measurement.

Specific capacitance from GCD spectrum is obtained by the following equation.

(3) $$C_{s}=\;Q/\left(\Delta V\;\times \;m\right)=\left(I\;\times \;\Delta t\right)/\left(\Delta V\;\times \;m\right)$$

Here I, Δt, ΔV and m represent the applied current, discharge time, accepted potential window of GCD curve, and active mass on substrates, respectively.

For Ragone plot, Power density and energy density were calculated from GCD curves by following equations:

(4) $$E=\left(C_{s}\times V_{\text{ma}x^{2}}\right)/\left(2\;\times \;3.6\right)$$

(5) P=(E×3600)/Δt

Where V_max_ is maximum voltage for GCD measurement, E is energy density in W h Kg^−1^ and P is power density in W Kg^−1^, respectively

**Fig. 7:**
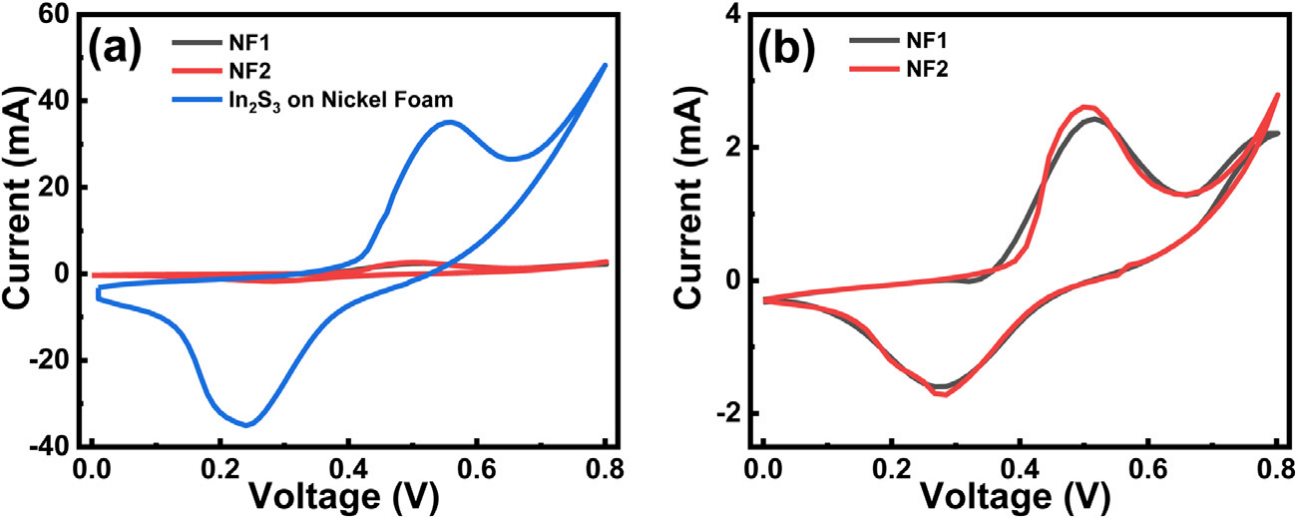
(a) Comparative 3T CV curves for bare Ni foams, Ni foam, processed without indium precursor, and directly grown In_2_S_3_ on Ni substrate. (b) Comparative 3T CV curves for NF1 and NF2.

We perform CV test at 5 mVs^−1^ (Fig. 7a and b) to evaluate the effect of nickel foam on capacitance performance and observed not much change in the enclosed area in sample NF1 and NF2. The existence of oxide/hydroxide impurity on nickel foam contributes to small redox reaction and capacitance [3]. Here NF1 (or NF2) stands for Ni foam without (or with) indium precursor, respectively.

**Fig. 8:**
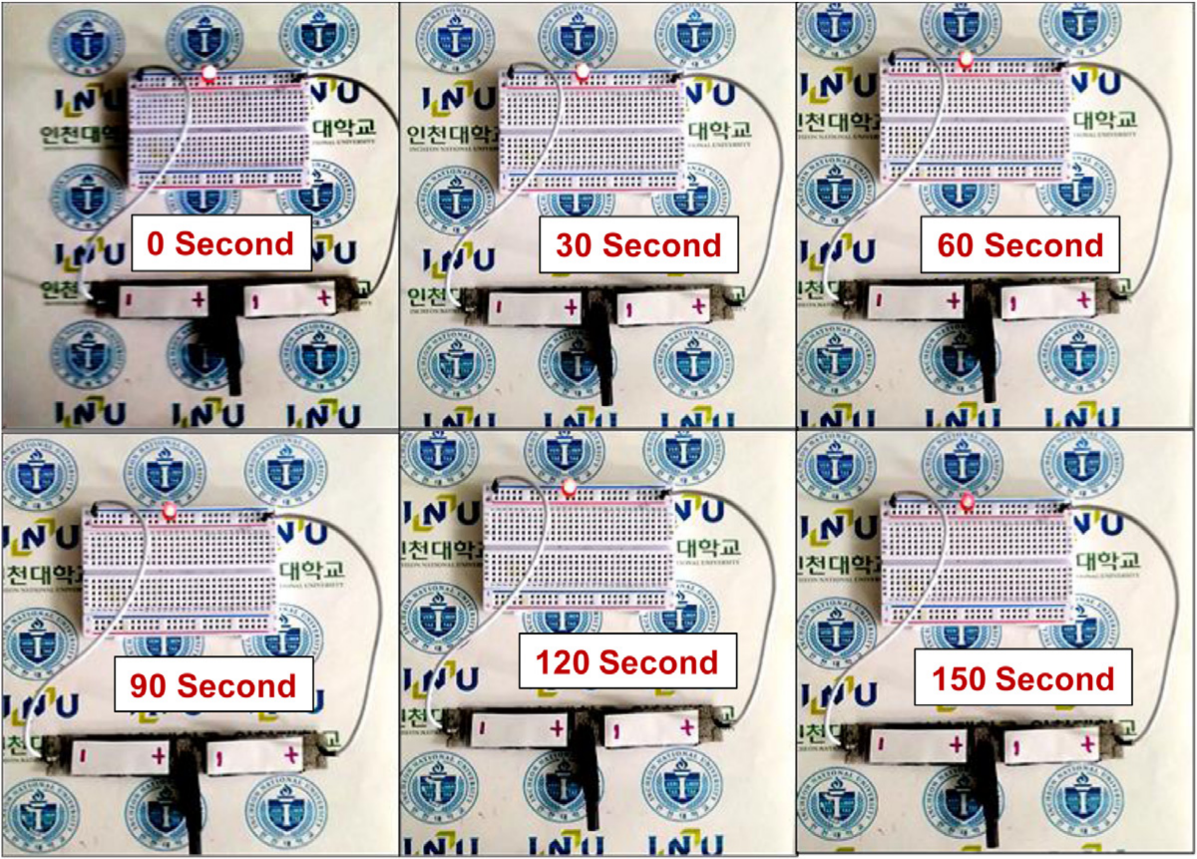
LED illumination with fabricated SSC device on different time frames (0 to 150 s).

**Fig. 9:**
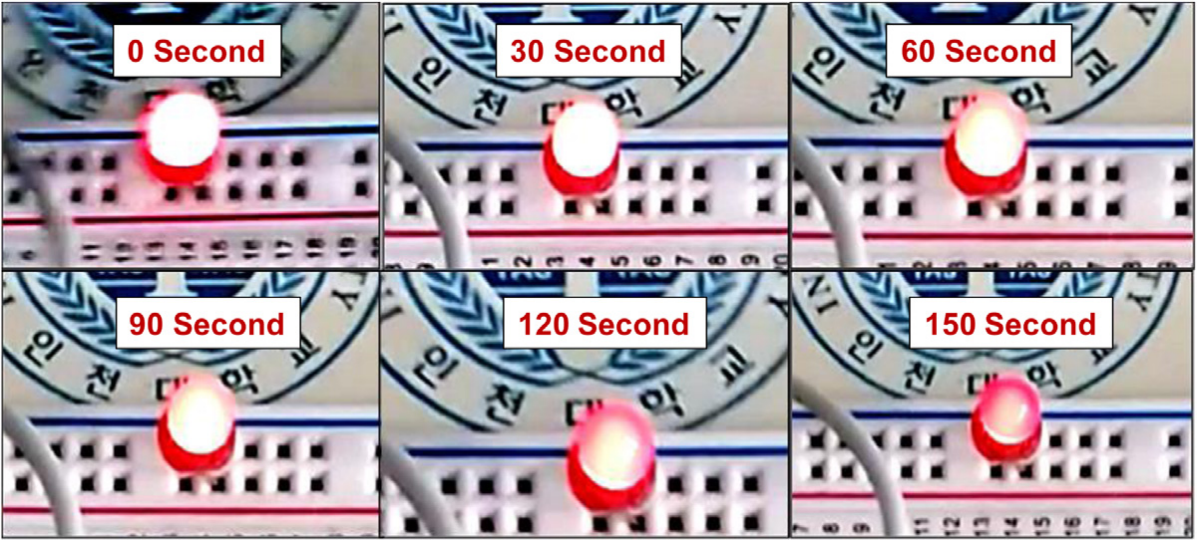
Magnified view of LED illumination with fabricated SSC device on different time frames (0 to 150 s).

Fig. 8 shows LED demonstration after small charging (2 mA current for 60 s charging) of two series-connected fabricated SSC devices with a different time frame (from 0 to 150 s). The result indicates, the fabricated device from prepared electrode material is highly efficient to illuminate LED for an adequately long time. The magnified image of LED is shown in Fig. 9.

Table 1 shows the estimated impedance parameters of electrode material by the fitting of that Nyquist curves. Impedance value after 3000 cycles are not much different from before cycle indicates the stable nature of synthesized material under frequency response.

**Table 1:** Estimated various impedance parameters from the Nyquist plot of prepared electrode materials.

Sample	R_s_ (Ω) Series Resistance	R_ct_ (Ω) Charge-Transfer Resistance	W (Ω) Warburg Resistance	C_p_ (F) Capacitance
Before 3000 Cycle	0.526	2.139	0.915	1.230
After 3000 Cycle	0.723	3.043	0.934	1.163

Table 2 is a comparative study of previous reported indium based electrode material on supercapacitor application and our current work to establish the suitability of our preparation material for energy storage application.

**Table 2:** Comparison of electrochemical characteristics of previous reported indium based electrode materials and current work.

Electrode Material	Morphology	Synthesis Method	Specific Capacitance	Cyclic stability (No. of Cycles)	Reference
In_2_O_3_	nanotowers, nanobouqets, nanocones, and nanowires	Chemical Vapor Deposition	16.6 mF cm^−2^	66.8 (1000)	[4]
In_2_O_3_	Thin Layer	Atomic Layer Deposition	1.36 mF/cm^2^	47.8 (2000)	[5]
Indium Tin Oxide	Nanowires	Magnetron Sputtering	956 F/g	–	[6]
Indium Oxide	Mesoporous Spheres	Hydrothermal	320 F/g	86 (3500)	[7]
In_2_O_3_/carbon	Aggregated Nanoparticles	Sol-Gel Approach	287 F/g	86 (5000)	[8]
In_2_O_3_/rGO	Aggregated Nanoparticles	Chemical Reaction	178.8 F/g	93.7 (5000)	[9]
In_2_O_3_	Nanodiscs	Hydrothermal	622 F/g	97 (10,000)	[10]
InP_3_	Layered	Liquid Phase Exfoliation	27.2 F cm^3^	88.7 (10,000)	[11]
In_2_S_3_	2D Layered	Solvothermal	897 Fg^−1^	90.81 (3000)	This Work

## 4 Experimental Design, Materials and Methods

### 4.1 Materials

Indium (III) chloride hydrate (Sigma–Aldrich), Thiourea (Sigma–Aldrich), polyvinyl alcohol (Sigma–Aldrich), and potassium hydroxide (Alfa Aesar) were used as without any further purification. Commercial porous nickel foams were used as conducting substrates for material deposition. Before use, NF (2 × 3 cm^2^) was thoroughly sonicated in 2 M HCl, acetone, and DI water for 10 min to remove extra oxides and dust from it.

### 4.2 Fabrication of SSC device

The symmetric solid-state supercapacitor (SSC) device was fabricated with prepared In_2_S_3_ electrode material in both electrodes. Nickel foam substrate electrode contact area is (2 × 1) cm^2^. Preparation of semi-solid electrolyte gel was done by adding 3 gram PVA and 3-gram KOH in 30 ml DI water. Then it was heated at 85˚ C with constant stirring until transparent gel formation. Two direct grown electrode material on NF were dipped in gel electrolyte for two minutes and then sandwiched parallel by keeping filter paper (Millipore - 0.45 µm) within as separator. After this, it is carefully covered by paraffin film to avoid leakage and short-circuiting and then kept overnight at room temperature to solidify it for further use.

### 4.3 Materials characterization

XRD Spectrum was acquired by X-ray Diffraction (XRD) - Rigaku SmartLab [Cu Kα radiation (λ = 1.54 A˚) for both powder and film samples deposited on nickel foam substrate. FTIR spectrum was examined from FT-IR Microscope: HYPERION 2000 for as-deposited material on NF substrate. TEM image, high-resolution TEM (HRTEM) image, atomic-resolution high-angle annular dark-field (HAADF) were recorded using an FEI/Talos F200X. The morphology, color mapping, and EDAX spectrum (Energy-dispersive X-ray spectroscopy) of prepared electrode material were analyzed by field emission scanning electron microscopy (FESEM) JEOL/JSM-7800F instrument. Specific surface area (Brunauere Emmette Teller (BET)) and pore size distribution (Barrete Joyere Halenda (BJH)) for prepared In_2_S_3_ powder material were characterized by using N2 adsorption-desorption isotherms by Tristar, ASAP 2020 / MICROMERITICS. All electrochemical studies were performed for (0.5 × 1 cm^2^) size electrode material. 1 M KOH liquid electrolyte solution was used for 3-electrode measurement (counter electrode- Pt wire and reference electrode- Ag/AgCl) by a Compactstat.h IVIUM Technologies electrochemical workstation. Active material mass on nickel foam substrate is 2.2 mg/cm^2^, which is calculated by mass difference method.

## Ethics Statement

Authors declare that the article is original and unpublished and is not being considered for publication elsewhere, and also it has not been submitted simultaneously anywhere. All authors have checked the revised manuscript and have agreed to the submission. The manuscript has been prepared according to the “Author Guidelines.”

## Declaration of Competing Interest

The authors declare that they have no known competing financial interests or personal relationships that could have appeared to influence the work reported in this paper. This work was supported by the Post-Doctor Research Program(2020) through Incheon National University (INU), Incheon, and Republic of Korea. This research was partly supported by Basic Science Research Program through the National Research Foundation of Korea(NRF) funded by the Ministry of Education (2019R111A1A0106362312)
